# Preparing Doctors to Secure Services Through an Empowering Buddy Programme Initiative: A Quality Improvement Project

**DOI:** 10.1192/bjo.2026.11508

**Published:** 2026-06-30

**Authors:** Ashal Uyanak

**Affiliations:** St Andrews Healthcare, Northampton, United Kingdom

## Abstract

**Aims:**

St Andrews Healthcare in Northampton is a tertiary psychiatric hospital with multiple divisions, aiming to lead Complex Mental Health care. It includes secure units across 3 divisions, in varying security levels and specialist needs.

Resident doctors identified through a survey that the current induction process does not support new doctors. Three main areas of concern were identified: information about their job roles, feeling confident for on-calls, and feeling welcomed. Some doctors commented that it took up to one year for them to feel comfortable in their duties.

**Methods:**

A Buddy programme was developed with formalised sessions to support a new non-consultant doctor. These included Speciality Doctors, Clinical Teaching Fellows and Associate Specialists and all eligible new doctors participated. It began as a 6-month pilot in Medium Secure Unit before launching to the whole of Northampton site. It ran between March 2024–December 2025. A comprehensive information booklet was created to support the Lead Buddies of each division on subjects to cover with their colleague. Feedback included pre-and post-intervention survey with binary answers, a Buddy feedback form, and additional feedback through e-mails. It was sponsored by one of the Associate Medical Directors and with the approval of all Clinical Directors.

**Results:**

The pre-programme survey noted only 18% of Resident doctor shaving enough information during their induction.

Post-intervention survey increased this to 100%. Confidence about their role has increased from 25% to 100%.

Despite the increase of confidence for starting on-calls, there was still remaining anxiety noted about the complexity. All doctors scored for anxiety about the on-calls in the pre-questionnaire. Post-intervention feedback noted remaining anxiety due to the size of the site. The programme enhanced the preparedness for on-calls by 57%.

Feeling welcomed had increased from 60% to 100% by the end of the programme.

**Conclusion:**

Buddy programme had an overwhelmingly positive effect for new Resident doctors. There are identified areas for future improvement. The organisational changes of the size of the site are likely to resolve one of these. The remaining areas include further analysis of the complexity of cases during on-calls and the turnover rate of non-consultant doctors.

